# Urinary Canthariasis Due to *Tenebrio molitor* Larva in a Ten-Year-Old Boy

**Published:** 2019-12-31

**Authors:** Mohammad Hassan Aelami, Alireza Khoei, Hamidreza Ghorbani, Farrokh Seilanian-Toosi, Elham Poustchi, Bibi Razieh Hosseini-Farash, Elham Moghaddas

**Affiliations:** 1 Department of Pediatrics and Hand Hygiene and Infection Control Research Center, Imam Reza Hospital, Mashhad University of medical Sciences, Mashhad, Iran; 2 Department of Pathology, Faculty of Medicine, Mashhad University of Medical Sciences, Mashhad, Iran; 3 Kidney Transplantation Complications Research Center, Mashhad University of Medical Sciences, Mashhad, Iran; 4 Department of Radiology, Faculty of Medicine, Mashhad University of Medical Sciences, Mashhad, Iran; 5 Department of Parasitology and Mycology, Faculty of Medicine, Mashhad University of Medical Sciences, Mashhad, Iran; 6 Cutaneous Leishmaniasis Research Center, Mashhad University of Medical Sciences, Mashhad, Iran

**Keywords:** Canthariasis, Bladder, Human, Child

## Abstract

Canthariasis is a human disease caused by infestation of beetle larvae. We report here an unusual cause of urogenital infection due to *Tenebrio molitor* in a 10-year-old boy suffering from severe and intermittent suprapubic pain from Nehbandan City, Northeastern Iran in 2018. After 9 months, three larvae were excreted. Keratinization of bladder wall was observed in histopathology. All laboratory evaluations were normal except for presence of microscopic hematuria. This report implicated that *T. molitor* could infest bladder accidentally and cause canthariasis and clinical symptoms that may lead to severe pain and bladder inflammation and hyperemia.

## Introduction

Canthariasis is a parasitic disease caused by beetle larvae, either humans or animals (1). Canthariasis in humans and animals due to *Tenebrio molitor* is an uncommon phenomenon. The reports are very rare and pathological effects are poorly known (2).

The first and only report on canthariasis due to *T. molitor* in bladder was reported about 375 years ago in a book entitled “Observations Medicae” (3). Moreover, *T. molitor* has invaded umbilicus and tonsils (4). However, the majority of reported cases of *T. molitor* larva in humans have been related to gastrointestinal tract (2, 5). Recently accidental ulcer infestation due *T. molitor* has been reported in a case with HIV/AIDS and skin ulcers (6).

*Tenebrio molitor* is a yellow mealworm commonly found as a stored-product pest. The life cycle of this organism comprises four stages including egg, larva, pupa and adult forms. The entire life cycle lasts approximately one year. Adult and the larvae feed on grains (hence the name mealworm), meat or decomposing animals including birds, spiders, rodents, lizards and some other beetles. Human is infected by the ingestion of eggs or larvae of *T. molitor* (7). *Tenebrio molitor* can also promote allergic reactions in exposed individuals (8).

Epidermolysis bullosa (EB) is the name for a group of rare genetic skin disorders that cause fragility in skin. Any trauma or friction to the skin can cause painful blisters (9).

In this case report, we describe an unusual case of canthariasis due to *T. molitor* in a 10-year-old boy with EB.

## Case presentation

**Fig. 1.**
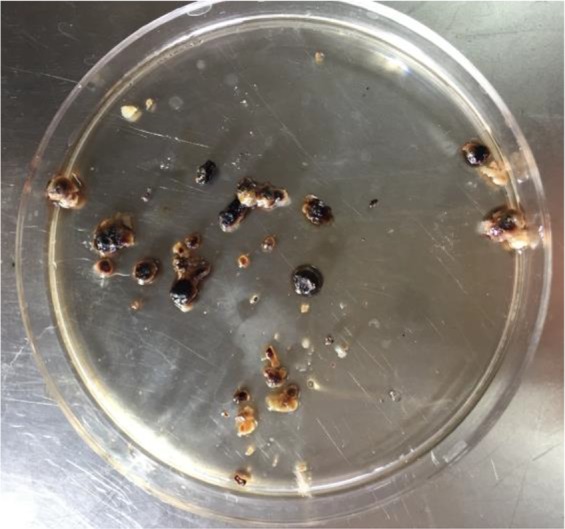
Brown sedimentations excreted via urethra

The patient was a 10-year-old boy (23kg) who had epidermolysis bullosa (EB) disorder referred from a local clinic from Nehbandan City in 2018, Northeastern of Iran. He suffered from periodic painful episodes in urinary system for the past 9 months. Symptoms were intermittent, and urine contained brown sediments similar to bladder stones (Fig. 1). There were no signs of gross hematuria and fever during the mentioned time. Complete medical examinations including urine analysis, urine culture, urine and blood biochemistry, hematology, Immunoassays for autoimmune diseases, thyroid function tests and renal ultrasound were performed.

Ultrasound was performed after observation of the excreted brown sediment from urethra for finding kidney stones. Kidney and bladder appeared normal in ultrasound. Urinalysis revealed no abnormality except for occult blood. Urinary culture was negative. Blood cell count and creatinine levels showed the normal ranges. Moreover, urine biochemistry was normal. Erythrocyte sedimentation rate (ESR) was 21 mm/1h.

**Fig. 2.**
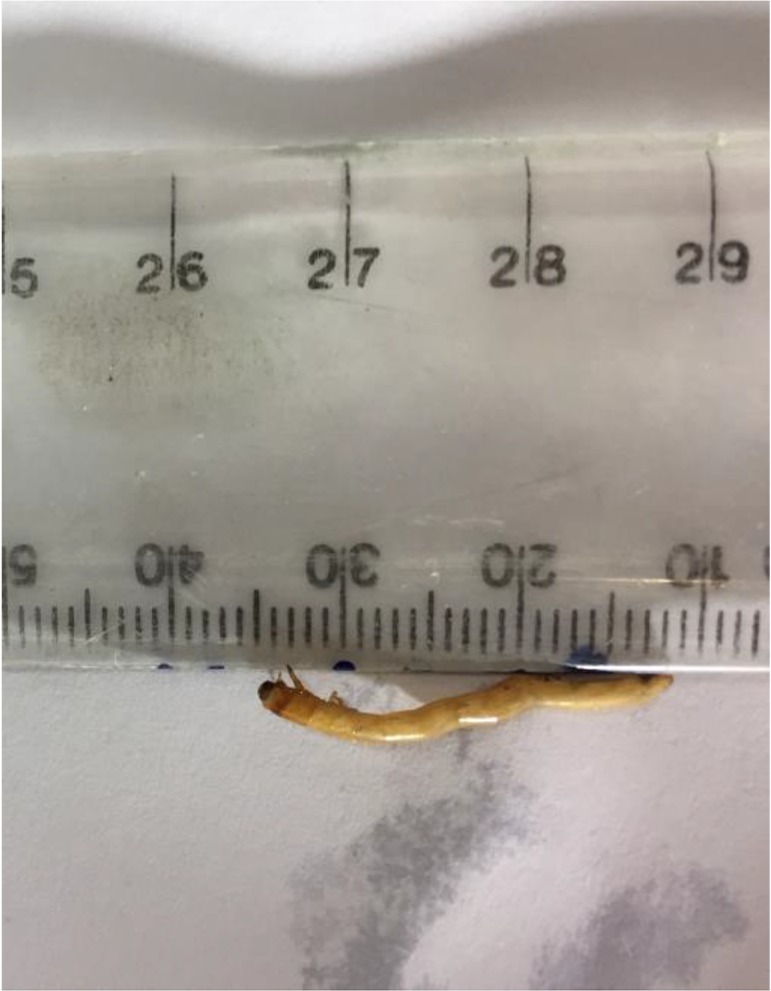
Macroscopic examination of the larva

The boy’s mother found three larvae in his urine more than two weeks after symptoms start. Morphologically, the larvae had six short legs close together near the head. The head has a pair of short hooks and was creamy white in color. The larvae had 26mm length and 0.5 mm width. They had three pairs of feet on the belly near the head and with each foot had 4 sections and nodes hocks curved claws (Fig. 2). Based on the characteristics, the larva was diagnosed as *T. molitor*.

**Fig. 3.**
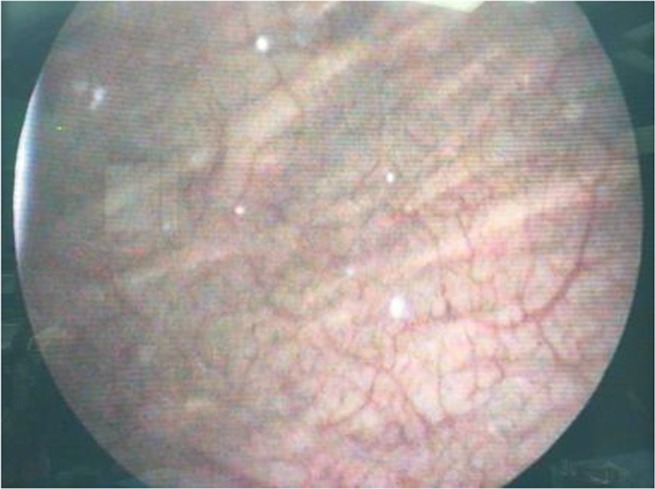
Cystoscopy finding including debris, hyperemia and inflammation

**Fig. 4.**
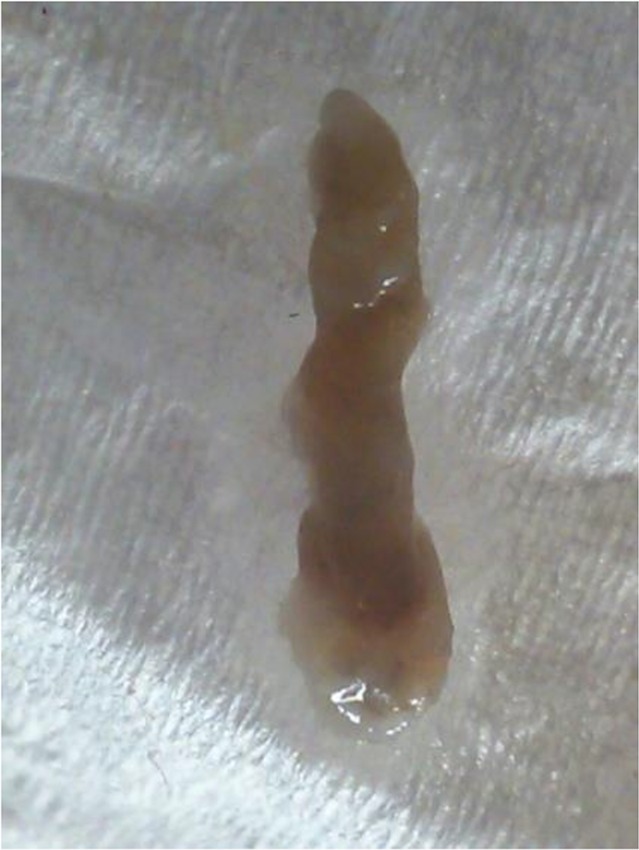
Disposal shell of insect after treatment by ivermectine

Debris, hyperemia and inflammation were identified in bladder and in the urethra cystoscopy (Fig. 3). However, no larva was seen in bladder and urethra. A single dose of 3mg of ivermectin was orally administrated. The patient recovered after oral Ivermectin therapy. Following treatment, high volume of insect shells was repulsed (Fig. 4). In addition, the abdominal pain completely resolved within a few months following treatment. Histopathology of excreted particles from urethral duct showed keratinizing squamous metaplasia after treatment.

Informed consent was taken from the patient’s parents. This study was reviewed and approved by the Ethics Committees of Mashhad University of Medical Sciences, Iran.

## Discussion

This case was initially misdiagnosed as kidney stones because the shedding of stone like substance without excretion of any larva from the urethra. Ultrasonography failed to detect the larva probably because the initial request was ordered for checking of kidney or urinary tract stones. Unlike most stones that are hard, the observed particles from this patient had soft structure. This beetle has 9–20 instars, the brown sedimentations were probably larvae shells during molting (https://en.wikipedia.org/wiki/Mealworm). On the other hand, we could speculate that the class of the scanner for determination of larvae was inadequate.

Beetle larvae have been recovered from human organs including tonsils (4), nose and bladder (10), umbilical cord (11), the gastrointestinal tract (4, 5), as well as from subcutaneous tissue in wild bird (1).

Canthariasis is a rare ectoparasitic condition. Among the reported cases, most of them were gastrointestinal. No previous cases in HIV/AIDS patients have been reported, neither associated with skin ulcers. Beetles could be found in houses where dried grains are stored, particularly this is case for *T. molitor*. Adult Tenebrio laid eggs on the skin of our patient. Females lay eggs and larvae develop within few weeks at necrotic tissue. In fact, scars attract the beetle and its larvae are fed on a variety of dried plant or animal matter and are known to scavenge on carcasses of dead animals.

Moreover, canthariasis has been reported in a case with HIV/AIDS patients associated with skin ulcer (6). Ulcers in HIV patients and other diseases related to skin damages could increase susceptibility to a wide range of infections such as canthariasis.

The present case had EB is a genetic disorder that result in easy blistering of the skin and mucous membranes. The patients with EB are susceptible to infection due to damage in skin physical barrier (9).

Beetles are a common household pest found in stored grain and stored food products. Adult of *T. molitor* laid eggs on the in or around the urogenital opening of our patient when he was resting. Then, the eggs hatch and larvae migrate along the urethra with consequent canthariasis. Some previous studies suggested this route of transmission for urinary myiasis (12,13)

This larva is unable to dwell in subsequently of the bladder wall. Because of this, our patient showed no macroscopic hematuria, and the bladder wall thickness was also normal (3mm). However, because of long stimulation time, wall bladder showed pathological changes. There were no lesion(s) in radiographic investigation.

*Tenebrio molitor* can be the intermediate hosts of the rat tapeworm, *Hymenolepis diminuta* (4). On the other hand, human is the accidental host of *H. diminuta* by ingestion of beetles or meal worms containing metacestode. Up to now, seven human patients have been reported from Iran to be infected with *H. diminuta* (14). Therefore, unintentional swallowing of *T. molitor* may be a possible route of transmission.

## Conclusion

Infection with *T. molitor* can present as an emergency condition with acute pain episodes in urinary system. Understanding of this infection should be considered when there are no evident signs for stones in urinary system in routine management.
